# Inhibition of DNM1L and mitochondrial fission attenuates inflammatory response in fibroblast‐like synoviocytes of rheumatoid arthritis

**DOI:** 10.1111/jcmm.14837

**Published:** 2019-11-21

**Authors:** Xiaoyan Wang, Zhufeng Chen, Xuemei Fan, Wei Li, Jiaqi Qu, Chuan Dong, Zhixue Wang, Zhenwei Ji, Yang Li

**Affiliations:** ^1^ Department of Rheumatology and Immunology The Second Affiliated Hospital of Harbin Medical University Harbin China; ^2^ Department of Orthopaedics Tangdu Hospital The Second Affiliated Hospital of Air Force Medical University Xi'an China

**Keywords:** dynamin 1‐like protein, fibroblast‐like synoviocyte, inflammation, mitochondrial fission, rheumatoid arthritis

## Abstract

Mitochondrial fission and fusion are important for mitochondrial function, and dynamin 1‐like protein (DNM1L) is a key regulator of mitochondrial fission. We investigated the effect of mitochondrial fission on mitochondrial function and inflammation in fibroblast‐like synoviocytes (FLSs) during rheumatoid arthritis (RA). DNM1L expression was determined in synovial tissues (STs) from RA and non‐RA patients. FLSs were isolated from STs and treated with a DNM1L inhibitor (mdivi‐1, mitochondrial division inhibitor 1) or transfected with *DNM1L*‐specific siRNA. Mitochondrial morphology, DNM1L expression, cell viability, mitochondrial membrane potential, reactive oxygen species (ROS), apoptosis, inflammatory cytokine expression and autophagy were examined. The impact of mdivi‐1 treatment on development and severity of collagen‐induced arthritis (CIA) was determined in mice. Up‐regulated DNM1L expression was associated with reduced mitochondrial length in STs from patients with RA and increased RA severity. Inhibition of DNM1L in FLSs triggered mitochondrial depolarization, mitochondrial elongation, decreased cell viability, production of ROS, IL‐8 and COX‐2, and increased apoptosis. DNM1L deficiency inhibited IL‐1β–mediated AKT/IKK activation, NF‐κBp65 nuclear translocation and LC3B‐related autophagy, but enhanced NFKBIA expression. Treatment of CIA mice with mdivi‐1 decreased disease severity by modulating inflammatory cytokine and ROS production. Our major results are that up‐regulated DNM1L and mitochondrial fission promoted survival, LC3B‐related autophagy and ROS production in FLSs, factors that lead to inflammation by regulating AKT/IKK/NFKBIA/NF‐κB signalling. Thus, inhibition of DNM1L may be a new strategy for treatment of RA.

## 1 INTRODUCTION

Rheumatoid arthritis (RA) is a chronic autoimmune inflammatory disease and is characterized by progressive destruction of multiple joints and synovial.^1^ Despite significant therapeutic advances, RA remains a huge threat for human health due to the high rate of patient disability. During the pathogenic process of RA, fibroblast‐like synoviocytes (FLSs) in the intimal lining can produce high levels of pro‐inflammatory cytokines and contribute to cartilage destruction.^2^ FLSs have been considered therapeutic targets for RA interventions. However, how FLSs contribute to the pathogenesis of RA has not been clarified.

Hypoxia can alter mitochondrial morphology and lead to mitochondrial dysfunctions, with reduced levels of adenosine triphosphate (ATP) production, excessive reactive oxygen species (ROS) production, increasing mitochondrial DNA mutations and oxidative damages, which in turn enhance inflammation in the joint, contributing to the pathogenesis of RA.^3^, ^4^, ^5^ Mitochondria are the main inducer and target of ROS.^6^ A previous study has shown that excessive ROS induces mitophagy,^7^ and feedback can remove damaged mitochondria to reduce ROS accumulation.^8^ ROS can activate the NF‐κB signalling to produce pro‐inflammatory cytokines, promoting synovial inflammation.^9^ It is notable that a defect in the mitochondrial respiratory chain (MRC) complex IV, and increased autophagy (mediated by LC3 and Beclin 1) are associated with the development of RA.^10^, ^11^ Inhibition of mitochondrial function can aggravate inflammatory responses in FLSs.^12^ Inhibition of mitochondrial functions exacerbates inflammatory responses in the joint in vivo.^13^ In addition, treatment with anti‐tumour necrosis factor (TNF) α can suppress mitochondrial mutagenesis in inflammatory arthritis.^14^ Mitochondrial membrane depolarization in peripheral blood lymphocytes has been reported in patients with RA.^15^ Accordingly, protecting mitochondrial function may be an effective intervention for RA.

Mitochondrial dynamics (fusion and fission) are crucial for multiple mitochondrial functions, including respiratory capacity, apoptosis, mitochondrial DNA stability, response to cellular stress and autophagy.^16^ Notably, dynamin 1‐like protein (DNM1L, a GTPase) and mitochondrial fission protein 1 (FIS1) regulate mitochondrial fission, whereas mitofusin 1 (MFN1), MFN2 and OPA1 regulate mitochondrial fusion.^17^ Previous studies have shown that increased mitochondrial fission can promote ROS production, leading to oxidative stress.^18^, ^19^, ^20^, ^21^, ^22^, ^23^ Hence, targeting mitochondrial fission may represent a therapeutic strategy to inhibit oxidative stress‐related diseases. DNM1L is conservatively expressed in the cytoplasm of many types of cells and is crucial for mitochondrial fission.^24^ Following its activation, DNM1L can be translocated into the mitochondrial outer membrane and bind to mitochondrial adaptor protein FIS1 to drive the process of mitochondrial fission.^24^ Accumulated evidence has shown that excessive expression of DNM1L is associated with development of neurodegenerative diseases, diabetes, tumours, kidney diseases, sepsis and cardiac diseases.^17^, ^25^, ^26^, ^27^, ^28^, ^29^, ^30^ However, it is still unclear whether excessive expression of DNM1L‐mediated mitochondrial fission is associated with the progression of RA.

In this study, DNM1L expression was examined in synovial tissues (STs) of RA and non‐RA patients. The effects of DNM1L deficiency on RA FLSs and in mice with collagen‐induced arthritis (CIA) were studied. The results indicated that up‐regulated DNM1L expression in STs was associated with RA severity, and DNM1L deficiency altered mitochondrial morphology and reduced ROS production, inflammatory responses, and autophagy in FLSs by down‐regulating the AKT/IKK/NFKBIA/NF‐κB signalling. Therefore, DNM1L may be a new therapeutic target, and inhibition of mitochondrial fission may be an effective intervention for RA.

## 2 MATERIALS AND METHODS

### 2.1 Reagents

The specific reagents used in this study included antibodies against DNM1L (A2586), GAPDH (AC002), AKT (A3145), IKK (A2087), NF‐kBp65 (A2547), IL‐8 (A2541) from ABclonal (Wuhan, China); antibodies against p‐AKT (4060), p‐IKK (2697T), LC3B (3868T), COX‐2 (12282T) from Cell Signaling Technology; antibodies against NFKBIA (10268‐1‐AP) from Proteintech; the DNM1L inhibitor Mdivi‐1, a mitochondrial division inhibitor 1 (M0199) from Sigma‐Aldrich; IL‐1β (200‐01B) from Peprotech; MitoTracker Green, BCA protein assay kit and Annexin V‐FITC (C1048, P0010S, C1063) from Beyotime; *DNM1L‐*specific small interfering RNA (siRNA) and control scramble siRNA from Guangzhou RiboBio; the Genomic DNA Removal Kit, SYBR Green PCR Kit from ABM; the CCK8 kit from MEC; ROS Fluorescent Probe‐DHE from Vigorous; primers from Sangon Biotech; and SDS‐PAGE gels from Epizyme.

### 2.2 Tissue collection and cell culture

Synovial tissues (STs) were obtained from patients with RA, who underwent a joint replacement (hit or knee) surgery in the Department of Orthopaedics of Tangdu Hospital, the Second Affiliated Hospital of Air Force Medical University from January 2018 to June 2018. Two RA markers (anti‐cyclic peptide containing citrulline [anti‐CCP] antibody, rheumatoid factor [RF]) and 2 inflammation markers (erythrocyte sedimentation rate [ESR], and high‐sensitivity C‐reactive protein [hs‐CRP]) were measured in each patient. Disease activity was calculated using the Disease Activity Score 28 joint count (DAS 28).^15^, ^31^, ^32^, ^33^ Control STs were obtained from patients with meniscus injuries without a history of acute or chronic arthritis, who underwent an arthroscopic surgery during the same period. There were four male and six female RA patients with ages ranging from 42 to 72 years (53.1 ± 11.53). There were three male control patients with ages ranging from 21 to 30 years (26.3 ± 4.75).

The partial fresh STs were washed with PBS and cut into small pieces. Subsequently, the tissue pieces were digested with 4 mg/mL of collagenase I (Invitrogen) at 37°C for 2 hours and filtered through a cell strainer (70 µm), followed by centrifugation. The obtained cells were cultured in DMEM/F12 supplemented with 10% fetal bovine serum (FBS), 100 U/mL of penicillin and 100 µg/mL of streptomycin. The cells at passages of 3‐6 were used as FLSs for experiments. All patients signed the written informed consent agreements before participation. The experimental protocols were approved by the Ethics Committee of Tangdu Hospital, the Second Affiliated Hospital of Air Force Medical University.

### 2.3 Immunohistochemistry (IHC)

Another part of STs was fixed in 10% formalin overnight and paraffin‐embedded. The ST sections were deparaffinized, rehydrated and treated with 3% H_2_O_2_ in methanol for 10 minutes, followed by antigen retrieval in a microwave. After being blocked with 5% BSA (AR0004; Boster), the sections were probed with anti‐DNM1L (1:300) at 4°C overnight, and the bound antibodies were detected with horseradish peroxidase (HRP)‐conjugated goat anti‐rabbit antibodies (1:1000, AS014; ABclonal), followed by visualization using diaminobenzidine. Images were obtained under a light microscope (Olympus).

### 2.4 Transfection of siRNA

Fibroblast‐like synoviocytes (1 × 10^5^ cells/mL) were cultured in 6‐well plates overnight and then transfected with 100 nmol/L *DNM1L*‐specific or control siRNA using lipofectamine 3000 (Invitrogen), according to the manufacturer's instruction. Three days later, DNM1L expression was determined by qRT‐PCR and Western blot assays.

### 2.5 Quantitative real‐time polymerase chain reaction (qRT‐PCR)

Fibroblast‐like synoviocytes (1 × 10^5^ cells/mL) were treated with vehicle control, 50 µmol/L mdivi‐1 or siRNA for 72 hours. Total RNA was extracted from the different groups of cells or fresh STs using TRIzol Reagent (Invitrogen). After removing genomic DNA using the Genomic DNA Removal Kit (ABM), each RNA sample (2 µg) was reversely transcribed into cDNA. The relative levels of target mRNA transcripts were determined by qRT‐PCR using the SYBR Green PCR Kit (ABM). Specific primers (Table 1) were used with the Rotor‐Gene Q RT‐PCR instrument (Qiagen GmbH, Hilden, Germany). All mRNA levels are expressed relative to the control (*GAPDH*) using the 2^−ΔΔCt^ method.^34^

**Table 1:** The sequences of primers

Human	
GAPDH	forward: 5′‐GGAGCGAGATCCCTCCAAAAT‐3′
	reverse: 5′‐GGCTGTTGTCATACTTCTCATGG‐3′
MFN1	forward: 5′‐TGGCTAAGAAGGCGATTACTGC‐3′
	reverse: 5′‐ TCTCCGAGATAGCACCTCACC‐3′
DNM1L	forward: 5′‐TAGCAGCAGTGACAGCGAGGA‐3′
	reverse: 5′‐GGGAGTAAGCCCTGAACCAAT‐3′
IL‐6	forward: 5′‐ ACTCACCTCTTCAGAACGAATTG‐3′
	reverse: 5′‐CCATCTTTGGAAGGTTCAGGTTG‐3′
IL‐8	forward: 5′‐ACTGAGAGTGATTGAGAGTGGAC‐3′
	reverse: 5′‐AACCCTCTGCACCCAGTTTTC‐3′
COX‐2	forward: 5′‐TTCAAATGAGATTGTGGGAAAATTGCT‐3′
	reverse: 5′‐AGTTCA TCTCTGCCTGAGTATCTT‐3′
MMP13	forward: 5′‐GACCCTGGAGCACTCATGT‐3′
	reverse: 5′‐ CATTTGTCTGGCGTTTTTGGAT‐3′
Mouse	
GAPDH	forward: 5′‐AGGCCGGTGCTGAGTATGTC‐3′,
	reverse: 5′‐TGCCTGCTTCACCACCTTCT;
NLRP3	forward: 5′‐CTGGCTGCGGATGGAATTTG‐3′
	reverse: 5′‐CTGGTCCTTTCCTCACGGTC‐3′
TNF‐α	forward: 5′‐ATGTCTCAGCCTCTTCTCATTC‐3′
	reverse: 5′‐GCTTGTCACTCGAATTTTGAGA‐3′
MMP13	forward: 5′‐CTTCCTGATGATGACGTTCAAG
	reverse: GTCACACTTCTCTGGTGTTTTG.
IL‐6	forward: 5′‐CTCCCAACAGACCTGTCTATAC‐3′
	reverse: 5′‐CCATTGCACAACTCTTTTCTCA‐3′
IL‐10	forward: 5′‐TTCTTTCAAACAAAGGACCAGC‐3
	reverse: 5′‐GCAACCCAAGTAACCCTTAAAG −3
COX‐2	forward: 5′‐GGTCATTGGTGGAGAGGTGTA‐3′
	reverse: 5′‐TGAGTCTGCTGGTTTGGAATAG‐3′

### 2.6 Transmission electron microscopy (TEM) and confocal microscopy

The different groups of FLSs and STs were fixed in glutaraldehyde at 4°C for 2 hours and post‐fixed in 1% citric acid‐0.1 mol/L phosphate buffer (PB) for 2 hours, followed by embedded. The ultra‐thin sections (70 nm) were stained with uranyl acetate and lead citrate. Images were collected using TEM (Hitachi, HT7700). In addition, the mitochondrial morphology of different groups of FLSs was examined by confocal microscopy (Zeiss 800) after staining with fluorescent MitoTracker Green, according to the manufacturer's instructions. In particular, cells (1 × 10^4^ cells/ well) were cultured in a chamber for 24 hours, stained with MitoTracker Green at 37°C in the dark for 40 minutes, and then examined under a confocal microscope. The different groups of cells were also stained with JC‐1 using the JC‐1 assay kit (Beyotime, C2006), according to the manufacturer's instruction. The fluorescent signals from this stain were also examined under a confocal microscope to determine mitochondrial membrane potential.

### 2.7 Western blot analysis

ST samples (100 mg wet‐weight/sample) were homogenized and centrifuged. FLSs (2.5 × 10^5^ cells/well) were transfected with *DNM1L*‐specific siRNA and control siRNA or treated with 50 µmol/L mdivi‐1 for 72 hours, followed by treatment with/without, IL‐1β (10 ng/mL) for 10 minutes. The different groups of cells were lysed with RIPA buffer containing proteinase and phosphatase inhibitors and centrifuged. The tissue homogenates or cell lysates (30 µg/lane) were separated by sodium dodecyl sulphate‐polyacrylamide gel electrophoresis (SDS‐PAGE) on 10% or 12.5% gels and electrophoretically transferred onto polyvinylidene difluoride (PVDF) membranes. The membranes were blocked with 5% fat‐free milk powder in TBST and probed with primary antibodies at 4°C overnight. After being washed, the bound antibodies were detected with HRP‐goat anti‐rabbit (1:2000) or antimouse (1:5000) for 1 hour at room temperature and visualized using the enhanced chemiluminescent reagents. The levels of each target protein relative to the loading control (GAPDH) were determined by densitometric analysis using ImageJ software. The primary antibodies were diluted by 1:1000 for DNM1L, IKK and LC3B; 1:10 000 for GAPDH, 1:500 for p‐AKT, p‐IKK, AKT, NFKBIA, COX‐2 and IL‐8.

### 2.8 Cell viability and apoptosis assays

The cell viability was measured using the CCK‐8 assay kit. Briefly, FLSs (1 × 10^4^ cells/well) were cultured in 96‐well plates overnight and treated in triplicate with vehicle or different concentrations (10, 20, 30, 40 and 50 µmol/L) of mdivi‐1 for 24 hours. During the last 2‐hours culture, 10 µL of CCK8 solution was added to each well. The absorbance at 450 nm was measured using a microplate reader.

The apoptosis of different groups of cells was determined by flow cytometry using the Annexin V‐FITC apoptosis Detection Kit. Briefly, FLSs (5 × 10^5^ cells/tube) were stained with 5 µL Annexin V‐FITC and 10 µL PI in the dark for 10 minutes at room temperature. The percentages of apoptotic cells were determined by flow cytometry (Epics XL‐MCL, Beckman). In addition, following transfection with *DNM1L*‐specific miRNA or control scramble siRNA for 72 hours, cells viability and apoptosis were determined.

### 2.9 Reactive oxygen species (ROS) detection

The contents of cellular ROS were determined using ROS fluorescent probe‐dihydroethidium (DHE), according to the manufacturer's protocols. Briefly, FLSs were reacted with DHE (20 µmol/L) at 37°C in the dark for 30 minutes and then stained with DAPI. The fluorescent signals were observed in an inverted fluorescent microscope (Olympus, IX‐53), with excitation at 480 nm and emission at 590 nm.

### 2.10 Immunofluorescence staining

The NF‐κBp65 nuclear accumulation was examined by immunofluorescence. Briefly, FLSs (0.5 × 10^4^ cells/well) were cultured in 24‐well culture plates and treated with vehicle or the DNM1L inhibitor for 24 hours. The cells were fixed with 4% paraformaldehyde and blocked with confining liquid (containing Triton X‐100 and 5% BSA). After being washed, the cells were probed with anti–NF‐κBp65 (1:50) at 4°C overnight, stained with Cy3‐conjugated secondary antibody (1:500), and then, the nuclei were stained using DAPI. The fluorescent signals were observed in an inverted fluorescence microscope.

### 2.11 GTPase activity assay

Different concentrations of mdivi‐1 (0, 10, 25 and 50 µmol/L) were incubated with DNM1L (human) recombinant protein (Abnova, P01) for 30 minutes at 4°C. A GTPase Assay Kit (Abnova, KA1610) was then used to determine the GTPase activity of DNM1L according to the manufacturer's protocol.

### 2.12 Induction of collagen‐induced arthritis (CIA) and mdivi‐1 treatment

Male DBA/1 mice (7‐week‐old) were obtained from Beijing Weitong Lihua Experimental Animal Technology Co and were housed in a specific pathogen‐free facility with free access to food and water. The mice were immunized with 200 µg bovine type II collagen (Chondrex, 20022) in 50% Freund's complete adjuvant (FCA) (Sigma, F5881) at the tail base. On day 21, the mice were injected intradermally with bovine type II collagen in 50% incomplete Freund's adjuvant (IFA) (Chondrex, 7002). On day 28, the mice were randomized and treated intraperitoneally with DMSO (vehicle, n = 7) or mdivi‐1 (0.2 mg/mouse, n = 7) every other day for two weeks. The animal study was approved by the Ethics Committee of Tangdu Hospital, The Second Affiliated Hospital of Air Force Medical University (Xi'an, China).

### 2.13 Clinical and histological assessment of arthritis

The mice were monitored for arthritis signs every other day post‐treatment. The severity of arthritis was scored from 0 to 4.^35^ The paw thickness of individual mice was measured using a Vernier caliper. On day 50, the mice were killed, and their knee joints, liver and renal tissues were collected and fixed in 10% formalin. The tissue sections (4 µm) were stained with haematoxylin and eosin (H&E) or toluidine blue. In addition, their STs were used for analysing the expression of cytokine mRNAs. The ROS contents in crystal synovial membrane sections were determined by DHE staining.^13^

### 2.14 Statistical analysis

Data are presented as the means ± SD of three or more separate experiments. The difference between two groups was analysed by Student's *t* test. Correlation was determined from Pearson's correlation coefficient. All statistical analyses were performed using the SPSS software 19.0 (SPSS). A *P‐value* below .05 was considered statistically significant.

## 3 RESULTS

### 3.1 Enhanced mitochondrial fission in STs of RA patients is correlated with disease severity

**Figure 1:**
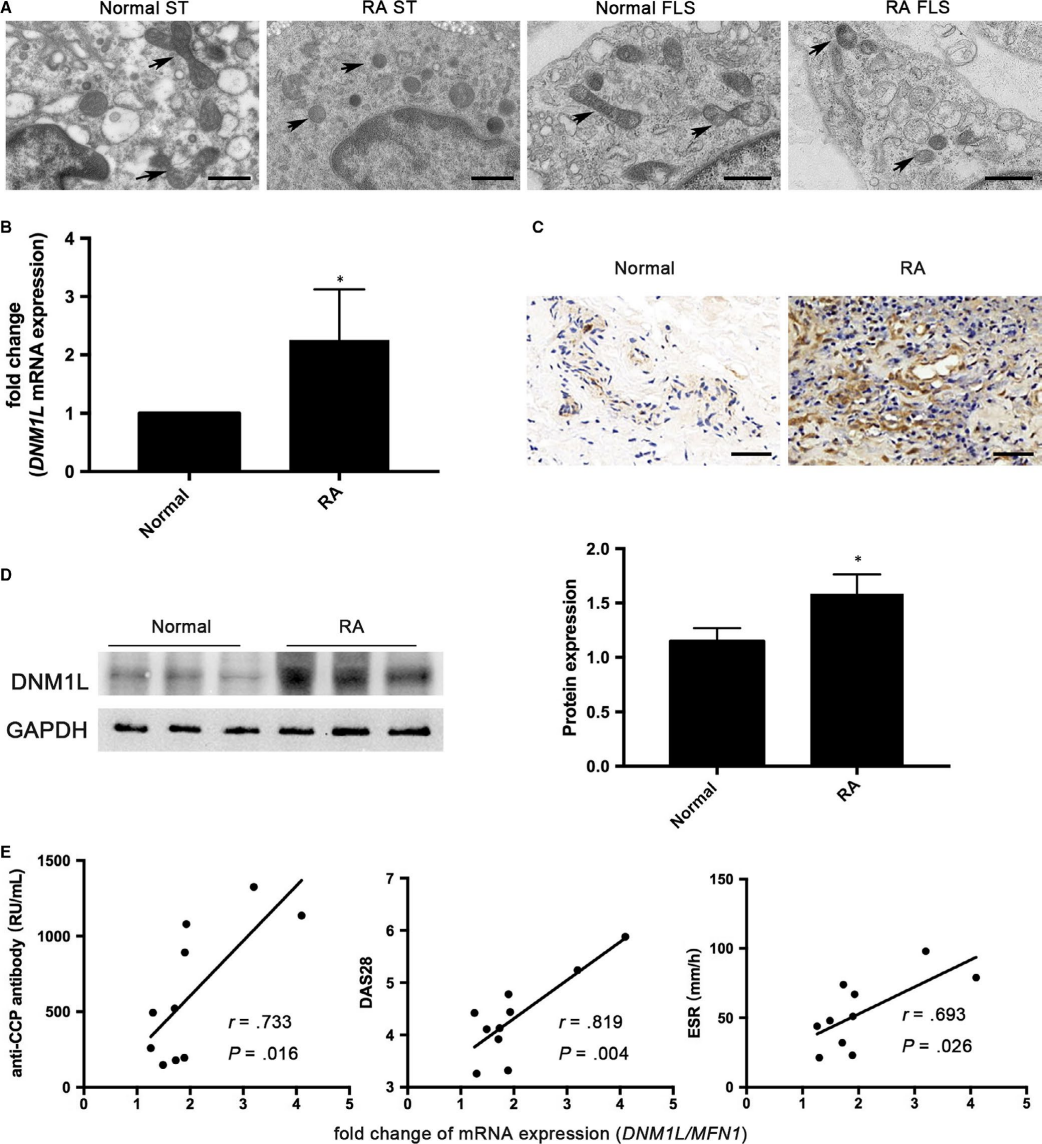
Enhanced mitochondrial fission in STs of RA patients correlates with disease severity. A, Representative TEM images of mitochondrial morphology in STs and FLS. Scale bars: 1 µm. B, qRT‐PCR analysis of *DNM1L* in STs. C, IHC analysis of DNM1L expression in STs. Scale bars: 50 µm. D, Western blot analysis of DNM1L in STs. E, Correlation of the ratio of *DNM1L*‐to‐*MFN1* with the level of serum anti‐CCP, DAS28 and ESR in RA patients. Data are the mean ± SD of each group. N = 10 RA patients and n = 3 non‐RA patients. *P < .05 vs the control

To investigate the mitochondrial dynamics in STs during the pathogenic process of RA, the morphologic changes of mitochondria were investigated in STs from RA and non‐RA patients and examined their FLSs by TEM. As shown in Figure 1A, mitochondrial length in the RA group was shorter than non‐RA group. Similarly, the length of mitochondria in FLSs from patients with RA was also shorter than that of FLSs from non‐RA patients. Furthermore, qRT‐PCR analysis indicated more *DNM1L* mRNA transcripts in the STs from the RA patients than non‐RA patients (Figure 1B). IHC and Western blot analysis exhibited that the levels of DNM1L expression in STs from patients with RA were remarkably up‐regulated, compared with that in the non‐RA patients (Figure 1C,D). Interestingly, the ratio of *DNM1L*‐to‐*MFN1* (determined by qRT‐PCR) had significantly positive correlations with the serum anti‐CCP level (*r* = .733, *P* = .016), DAS28 score (*r* = .089, *P* = .004), and ESR (*r* = .693, *P* = .026, Figure 1E). However, the *DNM1L*‐to‐*MFN1* ratio had no significant correlations with RF level, hs‐CRP level or disease duration (data not shown). In addition, there were no significant differences in the expression of *MFN2*, *FIS1* and *OPA1* mRNAs among RA and non‐RA individuals (data not shown). Hence, some markers of enhanced mitochondrial fission in the STs of RA patients correlated with disease severity.

### 3.2 DNM1L deficiency alters mitochondria morphology and mitochondrial membrane potential in FLSs

**Figure 2:**
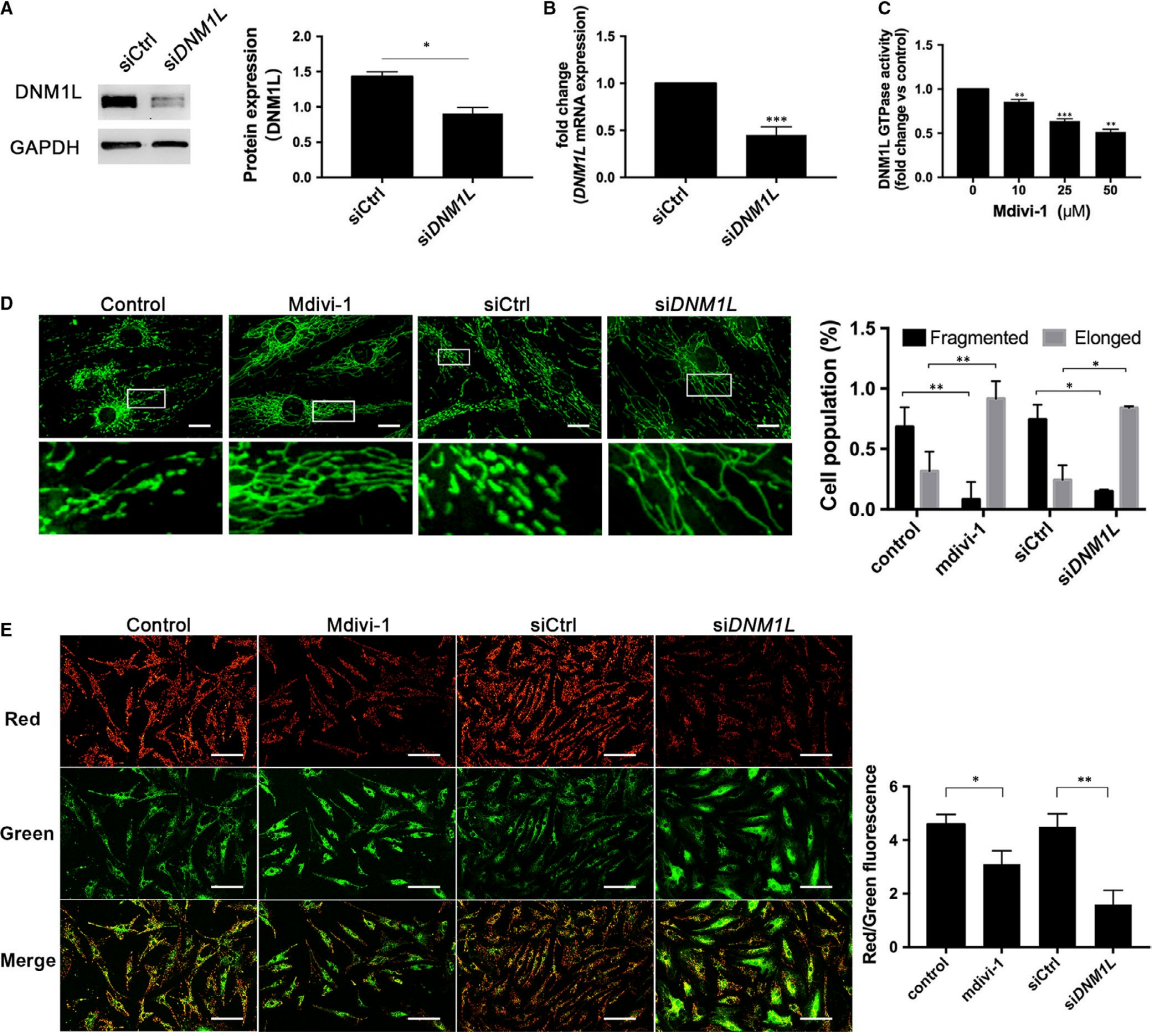
DNM1L deficiency alters mitochondria morphology and mitochondrial membrane potential in FLSs. (A, B) FLSs from RA patients were transfected with control (siCtrl) or *DNM1L*‐specific siRNA (si*DNM1L*) for 72 h, and the relative levels of DNM1L expression were determined by Western blot and qRT‐PCR. C, Effect of mdivi‐1 concentration on DNM1L GTPase activity. D, Representative confocal microscopy images of mitochondrial morphology in different groups of FLSs, with staining by MitoTracker Green and quantitation of these data. Scale bars: 20 µm. E, Representative confocal microscopy images of the effect of DNM1L deficiency on mitochondrial membrane depolarization (JC‐1 fluorescence) and quantitation of these data. The ratio of red to green signal was determined for quantitative analysis. Scale bars: 100 µm. Data are representative images or expressed as the mean ± SD of each group from three separate experiments. *P < .05; **P < .01; ***P < .001

DNM1L is crucial for mitochondrial fission. Next, we examined how DNM1L deficiency could affect mitochondrial morphology and membrane potential in FLSs. In comparison with the control cells transfected with scramble siRNA, transfection with *DNM1L*‐specific siRNA significantly decreased DNM1L protein expression by about 30% and *DNM1L* mRNA transcripts by 55% in FLSs (Figure 2A,B). We also measured GTPase activity of DNM1L after mdivi‐1 treatment. The results indicate that mdivi‐1 inhibited the GTPase activity of DNM1L in a dose‐dependent manner (Figure 2C). Following staining with MitoTracker Green, treatment of mdivi‐1 (50 µmol/L) or *DNM1L* silencing by transfection with *DNM1L*‐specific siRNA significantly increased the length of mitochondrial in FLSs (Figure 2D). Following JC‐1 staining, treatment of mdivi‐1 or *DNM1L* silencing reduced the ratios of red to green fluorescent signals, a hallmark of depolarization in FLSs (Figure 2E). Collectively, such data indicated that DNM1L deficiency altered mitochondrial morphology and induced mitochondrial membrane depolarization in FLSs.

### 3.3 DNM1L deficiency in FLSs reduces their viability and production of pro‐inflammatory cytokines and increases apoptosis

**Figure 3:**
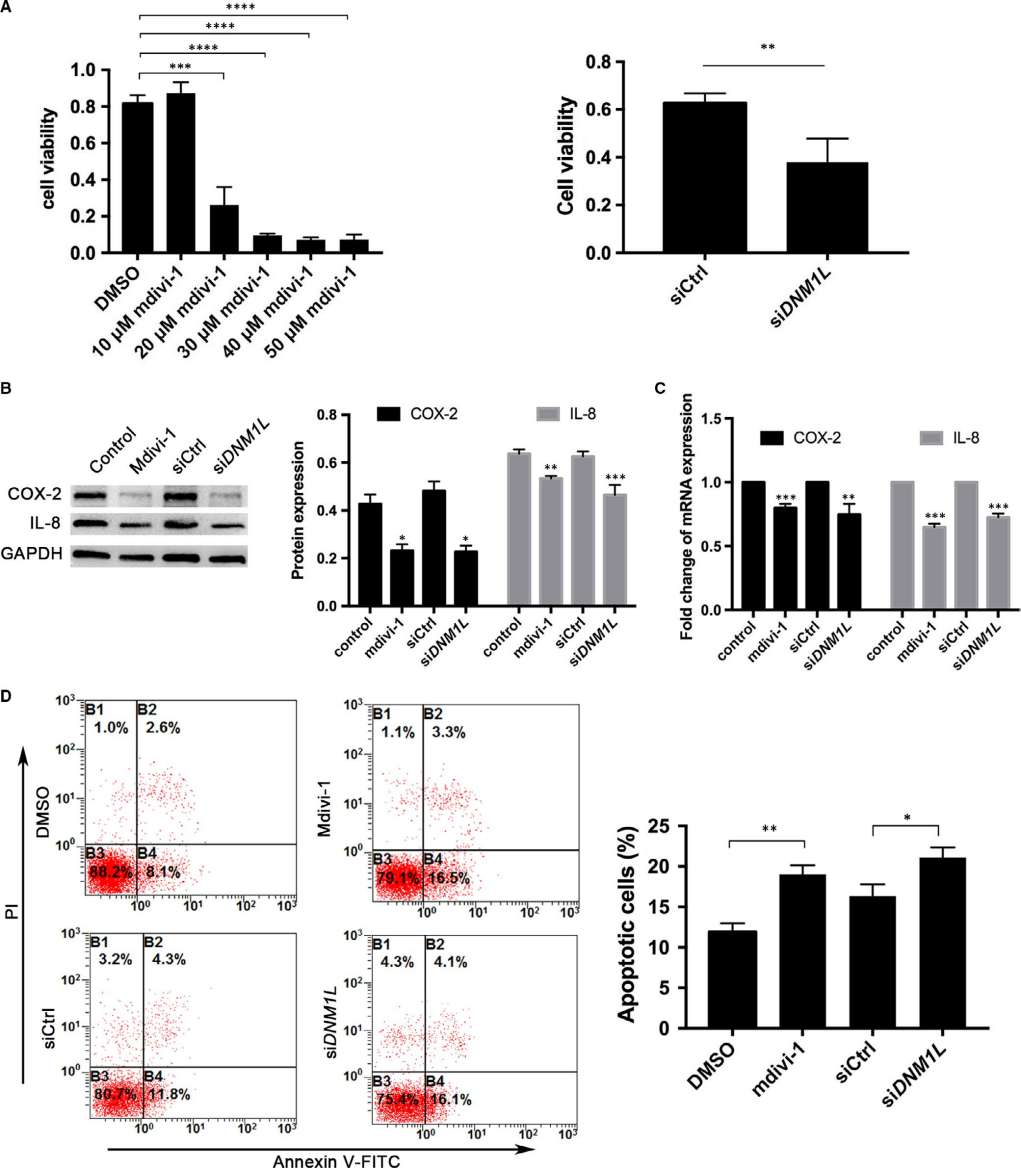
DNM1L deficiency in FLSs reduces their viability and production of pro‐inflammatory cytokines, and increases apoptosis. A, Cell viability was determined using the CCK‐8 assay. (B, C) Western blot and qRT‐PCR analyses of COX‐2 and IL‐8 expression in FLSs (mdivi‐1 concentration = 50 µmol/L). D, Representative flow cytometry data of apoptotic FLSs after staining with FITC‐Annexin V and PI, and quantitation of these data. Data are representative flow cytometry charts, images or expressed as mean ± SD of each group from three separate experiments. *P < .05; **P < .01; ***P < .001; ***P < .0001

Treatment with 10 µmol/L mdivi‐1 did not affect the viability of FLSs while treatment with 20‐50 µmol/L mdivi‐1 significantly reduced the viability of FLSs (Figure 3A). Similarly, *DNM1L* silencing also significantly reduced the viability of FLSs by nearly 45%. Furthermore, treatment with mdivi‐1 (50 µmol/L) or *DNM1L* silencing significantly decreased the relative levels of COX‐2 and IL‐8 expression in FLSs (Figure 3B,C). In addition, treatment with 50 µmol/L mdivi‐1 or DNM1L silencing significantly increased the percentages of apoptotic FLSs (Figure 3D). Thus, DNM1L deficiency reduced the viability of FLSs and their production of pro‐inflammatory cytokines by triggering apoptosis.

### 3.4 DNM1L deficiency reduces ROS production and LC3B‐related autophagy in FLSs

**Figure 4:**
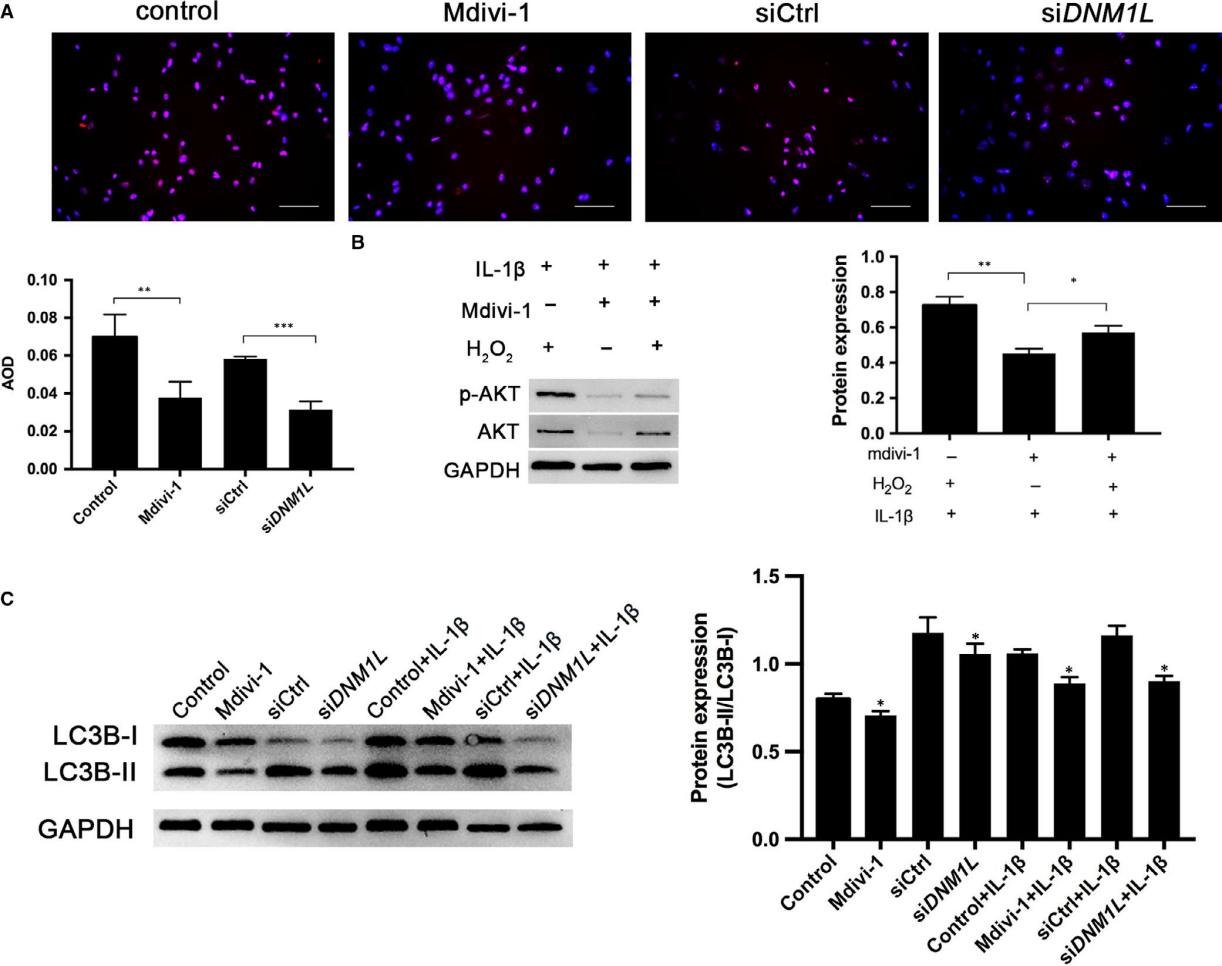
DNM1L deficiency reduces ROS production and autophagy in FLS. A, Representative DHE and DAPI staining, and quantitation of these data. AOD (average optical density) indicates the absolute absorption of DHE signal. Scale bars: 100 µm. B, Effect of mdivi‐1 on IL‐1β– and IL‐1β/H_2_O_2_–mediated AKT activation in FLSs. FLSs were treated with mdivi‐1 in the presence of IL‐1β for 10 min, and with or without, 100 µmol/L H_2_O_2_ for 12 h. C, Western blot analysis of LC3B‐I and LC3B‐II in the different groups of FLSs. Data are representative images or expressed as the mean ± SD of each group of cells from three separate experiments. *P < .05; **P < .01; ***P < .001

To investigate the impact of DNM1L deficiency on ROS production, FLSs were treated with mdivi‐1 (50 µmol/L) or transfected with *DNM1L*‐specific siRNA for 72 hours. The levels of intracellular ROS were measured by DHE staining. As expected, treatment with mdivi‐1 or *DNM1L* silencing significantly reduced the levels of ROS in FLSs (Figure 4A). Furthermore, while treatment with IL‐1β and H_2_O_2_ significantly induced AKT activation, treatment with mdivi‐1 abrogated the IL‐1β– and IL‐1β/H_2_O_2_–induced AKT expression and phosphorylation, although the inhibitory effect of mdivi‐1 on the IL‐1β/H_2_O_2_–induced AKT expression was less than that of IL‐1β–induced AKT activation in FLSs (Figure 4B). Moreover, treatment with mdivi‐1 or *DNM1L* silencing significantly decreased the ratio of LC3B‐II to LC3B‐I and the IL‐1β–increased ratios of LC3B‐II to LC3B‐I in FLSs (Figure 4C). The results suggest that ROS and LC3B‐related autophagy may play a key role in DNM1L‐mediated proliferation, inflammation and apoptosis in RA FLS.

### 3.5 DNM1L deficiency attenuates the IL‐1β–induced NF‐κB activation in FLSs

**Figure 5:**
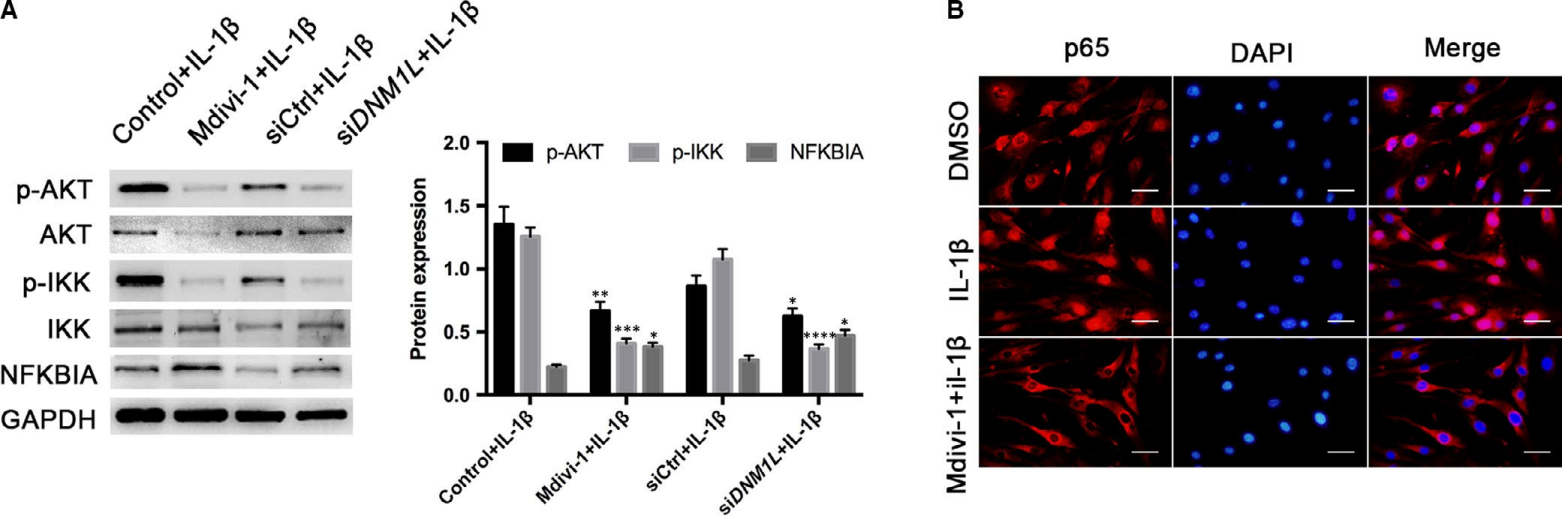
DNM1L deficiency attenuates the IL‐1β–induced NF‐κB activation in FLSs. A, The relative levels of AKT, IKK and NFKBIA expression and AKT and IKK phosphorylation were determined by Western blot. B, The nuclear translocation of NF‐κBp65 in the different groups of FLSs was determined by immunofluorescent microscopy. Scale bars: 50 µm. Data are representative images or expressed as the mean ± SD of each group of cells from three separate experiments. *P < .05; **P < .01; ***P < .001; ***P < .0001

To further explore the role of DNM1L in RA FLS inflammation, we examined the effect of DNM1L deficiency on the IL‐1β–induced AKT and IKK activation and NFKBIA expression in FLSs. We found that treatment with mdivi‐1 or *DNM1L* silencing mitigated the IL‐1β–induced AKT and IKK activation, but enhanced the IL‐1β–up‐regulated NFKBIA expression in FLSs (Figure 5A). Further immunofluorescence studies revealed that treatment with mdivi‐1 attenuated the IL‐1β–induced NF‐κBp65 nuclear translocation in FLSs (Figure 5B). Together, such data indicated that DNM1L deficiency attenuated the IL‐1β–induced NF‐κB activation in FLSs by down‐regulating AKT/IKK/NFKBIA signalling.

### 3.6 Treatment with mdivi‐1 ameliorates the severity of CIA in mice

**Figure 6:**
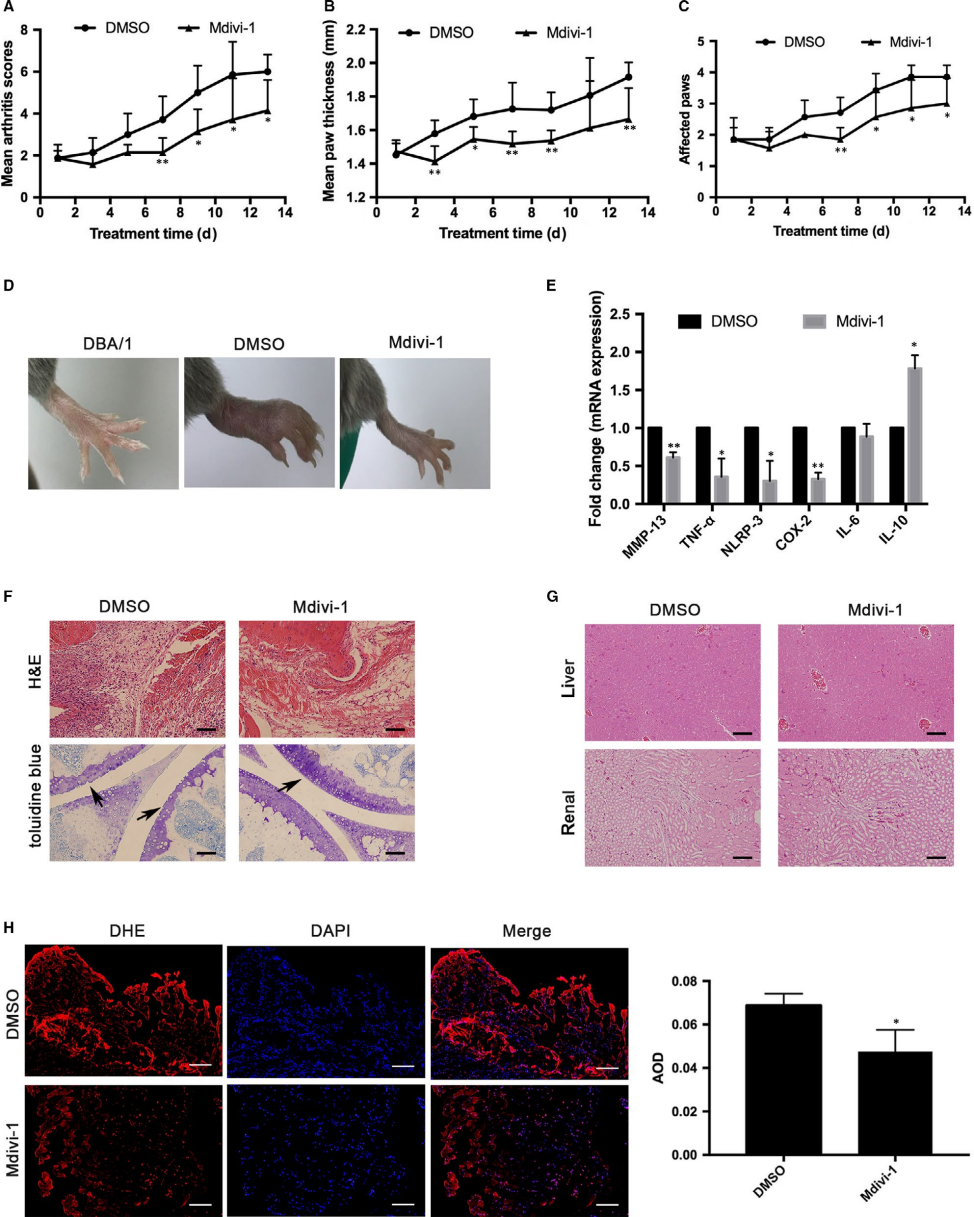
Treatment with mdivi‐1 ameliorates the severity of CIA in mice. After established CIA, the mice were randomized and treated with DMSO or 0.2 mg mdivi‐1 every other day for 2 wk. (A‐C) Their CIA clinical scores, paw thickness and affected paws were measured. D, Representative images of paws in the different groups of mice. E, The relative levels of inflammatory molecules in the joints of different groups of mice were determined by qRT‐PCR. F, Histological examination of the hind paws after H&E or toluidine blue staining. Scale bar: 100 µm. G, Histological examination of the liver and kidney tissues after H&E staining. Scale bar: 100 µm. H, DHE staining of ROS contents in the crystal synovial membranes of mice. Scale bar: 200 µm. Data are representative images or expressed as the mean ± SD of each group of mice (n = 7). *P < .05; **P < .01

Finally, we examined the potential therapeutic effect of mdivi‐1 treatment on the development and severity of CIA in mice. Compared with the control (DMSO) CIA mice, treatment with mdivi‐1 (0.2 mg/mouse) significantly decreased the clinical scores, paw thickness and affected paws and ameliorated the overt symptoms (Figure 6A‐D). Furthermore, treatment with mdivi‐1 significantly mitigated the CIA‐mediated upregulation of *MMP‐13, NLRP3, TNF‐α and COX‐2,* but not *IL‐6*, and increased the expression of *IL‐10* in the STs (Figure 6E). While control CIA mice displayed joint damages, treatment with mdivi‐1 preserved the intact joint architecture (Figure 6F). In addition, treatment with mdivi‐1 also decreased ROS production in the STs of mice (Figure 6H). However, histological examination revealed that treatment with mdivi‐1 did not change the morphology of the liver and kidney tissues in mice (Figure5G). Therefore, treatment with mdivi‐1 to impaired DNM1L activity and ameliorated the severity of CIA in mice by modulating the pro‐inflammatory and anti‐inflammatory responses, but had no effect on non‐target tissues (liver and kidney).

## 4 DISCUSSION

In the present study, we found that up‐regulated DNM1L expression in STs from patients with RA was associated with the severity of RA. Such findings support the notion that up‐regulated mitochondrial fission is associated with the development of different types of diseases, including inflammatory diseases.^4^, ^17^, ^25^, ^26^, ^27^, ^28^, ^29^, ^36^, ^37^, ^38^, ^39^, ^40^ To the best of our knowledge, this was the first report on up‐regulated DNM1L expression in STs from patients with RA and the correlations suggest that DNM1L expression may be a new biomarker to evaluate the severity of RA.

DNM1L is crucial for mitochondrial fission. *MFN1* knockout mice display fragmented mitochondria.^41^ In this study, we found that up‐regulated DNM1L expression was associated with decreased length of mitochondria in STs, consistent with previous observations.^4^, ^17^, ^42^ Similarly, *DNM1L* silencing and treatment with mdivi‐1 also increased the length of mitochondria in FSLs. Hence, up‐regulated DNM1L expression reflected enhanced mitochondrial fission, which should promote mitochondrial membrane depolarization and in tune enhance ROS production. Actually, a previous study has shown that mitochondrial membrane depolarization in peripheral lymphocytes is positively correlated with serum CRP levels and DAS28 values in patients with RA.^15^ DNM1L deficiency promoted mitochondrial membrane depolarization and decreased the levels of ROS and pro‐inflammatory COX‐2 and IL‐8 in RA FLSs, similar to that in synoviocytes.^12^ We also found that the ratio of *DNM1L‐*to‐*MFN1* transcripts correlated with the level of serum anti‐CCP antibodies, DAS28 and ESR values in patients with RA. Such data suggest that DNM1L‐related mitochondrial fission may contribute to the pathogenesis of RA.

Mdivi‐1 inhibits DNM1L GTPase activity and is a potent inhibitor of mitochondrial fission. Previous studies demonstrated that mdivi‐1 can inhibit myocardial infarction, stroke, neurodegenerative diseases, and cancers.^22^, ^25^, ^26^, ^36^, ^43^, ^44^, ^45^, ^46^ In the present study, we found that mdivi‐1 inhibited DNM1L GTPase activity in a dose‐dependent manner, consistent with previous studies.^43^, ^47^ Furthermore, treatment with mdivi‐1 significantly reduced the severity of CIA in mice by decreasing pro‐inflammatory cytokine production, but increasing anti‐inflammatory IL‐10 production. These, together with its inhibition on ROS and pro‐inflammatory COX‐2 and IL‐8 production in FLSs, suggest that inhibition of mitochondrial fission may modulate the balance of pro‐inflammatory and anti‐inflammatory responses in joint lesions. Given that mitochondria are crucial for nutritional metabolisms, it is possible that inhibition of mitochondrial fission may differentially modulate glucose and lipid metabolisms to affect pathogenic and regulatory T‐cell responses during the pathogenic process of RA.

It is well known that mitochondrial dysfunction can induce excessive ROS production, which promotes autophagy^17^ and exacerbates synovial inflammation by activating the NF‐κB signalling to promote the expression of pro‐inflammatory cytokines.^12^, ^48^ Furthermore, mitochondrial dysfunction can activate PI3K/AKT signalling, which is crucial for inflammation in RA FLSs.^17^, ^49^ In addition, the activated AKT can induce IKK phosphorylation and NF‐κB, which is down‐regulated by NFKBIA.^50^, ^51^ In this study, we found that treatment with mdivi‐1 or *DNM1L* silencing effectively decreased ROS production and LC3B‐related autophagy in FLSs, extending the previous observation in osteoblasts.^42^ Moreover, DNM1L deficiency also mitigated the IL‐1β–induced AKT and IKK phosphorylation and NF‐κBp65 nuclear translocation, but increased NFKBIA expression in RA FLS. Therefore, anti‐oxidants and inhibition of mitochondrial fission to attenuate the AKT/IKK/NF‐κB signalling may be promising strategies for treatment of RA.

This study had limitations, including a small sample size and the lack of studies on how inhibition of mitochondrial fission alters mitochondrial DNA and glucose and lipid metabolisms that affect pathogenic and regulatory T‐cell responses. Hence, future investigations in a bigger population are warranted to determine molecular mechanisms by which the mitochondrial dynamic factors regulate the pathogenesis of RA.

In conclusion, our results indicated that up‐regulated DNM1L expression in STs was associated with the severity of RA. Treatment with mdivi‐1 or *DNM1L* silencing altered mitochondrial morphology, promoted mitochondrial membrane depolarization and apoptosis, and decreased cell viability, ROS, LC3B‐related autophagy and pro‐inflammatory cytokine production in FLSs. The same treatment also mitigated the IL‐1β–mediated LC3B‐related autophagy and activation of AKT, IKK and NF‐κB in FLSs by up‐regulating NFKBIA expression. Treatment with mdivi‐1 significantly reduced the severity of CIA in mice by decreased ROS and modulating the balance of pro‐inflammatory and anti‐inflammatory responses. Therefore, mitochondrial fission may be new target for treatment of RA.

## CONFLICT OF INTEREST

The authors declare no competing interests.

## AUTHOR CONTRIBUTIONS

Wang Xiaoyan and Chen Zhufeng performed the research and wrote the paper. Fan Xuemei, Qu Jiaqi and Li Wei analysed the data. Dong Chuan, Wang Zhixue and Ji Zhenwei provided samples in the study. Li Yang and Wang Xiaoyan designed the research study.

## Data Availability

All data generated or analysed during this study are included in this article.

## References

[1] McInnes IB , Schett G . The pathogenesis of Rheumatoid Arthritis. N Engl J Med. 2011;365:2205‐2219. 22150039 10.1056/NEJMra1004965

[2] Bartok B , Firestein GS . Fibroblast‐like synoviocytes: key effector cells in rheumatoid arthritis. Immunol Rev. 2010;233:233‐255. 20193003 10.1111/j.0105-2896.2009.00859.x PMC2913689

[3] Ng CT , Biniecka M , Kennedy A , et al. Synovial tissue hypoxia and inflammation in vivo. Ann Rheumat Dis. 2010;69:1389‐1395. 20439288 10.1136/ard.2009.119776 PMC2946116

[4] Luo F , Herrup K , Qi X , Yang Y . Inhibition of Drp1 hyper‐activation is protective in animal models of experimental multiple sclerosis. Exp Neurol. 2017;292:21‐34. 28238799 10.1016/j.expneurol.2017.02.015 PMC5484055

[5] Stuart JA , Brown MF . Mitochondrial DNA maintenance and bioenergetics. Biochim Biophys Acta. 2006;1757:79‐89. 16473322 10.1016/j.bbabio.2006.01.003

[6] Balaban RS , Nemoto S , Finkel T . Mitochondria, oxidants, and aging. Cell. 2005;120:483‐495. 15734681 10.1016/j.cell.2005.02.001

[7] Kubli DA , Gustafsson AB . Mitochondria and mitophagy: the yin and yang of cell death control. Circ Res. 2012;111:1208‐1221. 23065344 10.1161/CIRCRESAHA.112.265819 PMC3538875

[8] Yu W , Fu YC , Wang W . Cellular and molecular effects of resveratrol in health and disease. J Cell Biochem. 2012;113:752‐759. 22065601 10.1002/jcb.23431

[9] Zhang J , Song X , Cao W , et al. Autophagy and mitochondrial dysfunction in adjuvant‐arthritis rats treatment with resveratrol. Sci Rep. 2016;6:32928. 27611176 10.1038/srep32928 PMC5017199

[10] Biniecka M , Fox E , Gao W , et al. Hypoxia induces mitochondrial mutagenesis and dysfunction in inflammatory arthritis. Arthritis Rheumat. 2011;63:2172‐2182. 21484771 10.1002/art.30395

[11] Xu K , Xu P , Yao JF , Zhang YG , Hou WK , Lu SM . Reduced apoptosis correlates with enhanced autophagy in synovial tissues of rheumatoid arthritis. Inflamm Res. 2013;62:229‐237. 23178792 10.1007/s00011-012-0572-1

[12] Valcarcel‐Ares MN , Riveiro‐Naveira RR , Vaamonde‐Garcia C , et al. Mitochondrial dysfunction promotes and aggravates the inflammatory response in normal human synoviocytes. Rheumatology. 2014;53:1332‐1343. 24609059 10.1093/rheumatology/keu016

[13] Vaamonde‐Garcia C , Loureiro J , Valcarcel‐Ares MN , et al. The mitochondrial inhibitor oligomycin induces an inflammatory response in the rat knee joint. BMC Musculoskelet Disord. 2017;18:254. 28606072 10.1186/s12891-017-1621-2 PMC5469149

[14] Biniecka M , Kennedy A , Ng CT , et al. Successful tumour necrosis factor (TNF) blocking therapy suppresses oxidative stress and hypoxia‐induced mitochondrial mutagenesis in inflammatory arthritis. Arthritis Res Ther. 2011;13:R121. 21787418 10.1186/ar3424 PMC3239359

[15] Moodley D , Mody G , Patel N , Chuturgoon AA . Mitochondrial depolarisation and oxidative stress in rheumatoid arthritis patients. Clin Biochem. 2008;41:1396‐1401. 18789914 10.1016/j.clinbiochem.2008.08.072

[16] Chan DC . Mitochondria: dynamic organelles in disease, aging, and development. Cell. 2006;125:1241‐1252. 16814712 10.1016/j.cell.2006.06.010

[17] Huang Q , Zhan L , Cao H , et al. Increased mitochondrial fission promotes autophagy and hepatocellular carcinoma cell survival through the ROS‐modulated coordinated regulation of the NFKB and TP53 pathways. Autophagy. 2016;12:999‐1014. 27124102 10.1080/15548627.2016.1166318 PMC4922447

[18] Vega RB , Horton JL , Kelly DP . Maintaining ancient organelles: mitochondrial biogenesis and maturation. Circ Res. 2015;116:1820‐1834. 25999422 10.1161/CIRCRESAHA.116.305420 PMC4443496

[19] Fan X , Hussien R , Brooks GA . H2O2‐induced mitochondrial fragmentation in C2C12 myocytes. Free Radic Biol Med. 2010;49:1646‐1654. 20801212 10.1016/j.freeradbiomed.2010.08.024 PMC2970628

[20] Jendrach M , Mai S , Pohl S , Voth M , Bereiter‐Hahn J . Short‐ and long‐term alterations of mitochondrial morphology, dynamics and mtDNA after transient oxidative stress. Mitochondrion. 2008;8:293‐304. 18602028 10.1016/j.mito.2008.06.001

[21] Wu S , Zhou F , Zhang Z , Xing D . Mitochondrial oxidative stress causes mitochondrial fragmentation via differential modulation of mitochondrial fission‐fusion proteins. FEBS J. 2011;278:941‐954. 21232014 10.1111/j.1742-4658.2011.08010.x

[22] Gan X , Huang S , Wu L , et al. Inhibition of ERK‐DLP1 signaling and mitochondrial division alleviates mitochondrial dysfunction in Alzheimer's disease cybrid cell. Biochim Biophys Acta. 2014;1842:220‐231. 24252614 10.1016/j.bbadis.2013.11.009 PMC3991235

[23] Gan X , Wu L , Huang S , et al. Oxidative stress‐mediated activation of extracellular signal‐regulated kinase contributes to mild cognitive impairment‐related mitochondrial dysfunction. Free Radic Biol Med. 2014;75:230‐240. 25064321 10.1016/j.freeradbiomed.2014.07.021 PMC4392773

[24] Westermann B . Mitochondrial fusion and fission in cell life and death. Nat Rev Mol Cell Biol. 2010;11:872‐884. 21102612 10.1038/nrm3013

[25] Rehman J , Zhang HJ , Toth PT , et al. Inhibition of mitochondrial fission prevents cell cycle progression in lung cancer. FASEB J. 2012;26:2175‐2186. 22321727 10.1096/fj.11-196543 PMC3336787

[26] Zhao J , Zhang J , Yu M , et al. Mitochondrial dynamics regulates migration and invasion of breast cancer cells. Oncogene. 2013;32:4814‐4824. 23128392 10.1038/onc.2012.494 PMC3911914

[27] Pal AD , Basak NP , Banerjee AS , Banerjee S . Epstein‐Barr virus latent membrane protein‐2A alters mitochondrial dynamics promoting cellular migration mediated by Notch signaling pathway. Carcinogenesis. 2014;35:1592‐1601. 24632494 10.1093/carcin/bgu069

[28] Wan YY , Zhang JF , Yang ZJ , et al. Involvement of Drp1 in hypoxia‐induced migration of human glioblastoma U251 cells. Oncol Rep. 2014;32:619‐626. 24899388 10.3892/or.2014.3235

[29] Otera H , Ishihara N , Mihara K . New insights into the function and regulation of mitochondrial fission. Biochim Biophys Acta. 2013;1833:1256‐1268. 23434681 10.1016/j.bbamcr.2013.02.002

[30] Huang S , Wang Y , Gan X , et al. Drp1‐mediated mitochondrial abnormalities link to synaptic injury in diabetes model. Diabetes. 2015;64:1728‐1742. 25412623 10.2337/db14-0758 PMC4407851

[31] Veale DJ , Orr C , Fearon U . Cellular and molecular perspectives in rheumatoid arthritis. Semin Immunopathol. 2017;39:343‐354. 28508153 10.1007/s00281-017-0633-1

[32] Rantapaa‐Dahlqvist S , de Jong BA , Berglin E , et al. Antibodies against cyclic citrullinated peptide and IgA rheumatoid factor predict the development of rheumatoid arthritis. Arthritis Rheum. 2003;48:2741‐2749. 14558078 10.1002/art.11223

[33] Nielen MM , van Schaardenburg D , Reesink HW , et al. Specific autoantibodies precede the symptoms of rheumatoid arthritis: a study of serial measurements in blood donors. Arthritis Rheum. 2004;50:380‐386. 14872479 10.1002/art.20018

[34] Livak KJ , Schmittgen TD . Analysis of relative gene expression data using real‐time quantitative PCR and the 2(‐Delta Delta C(T)) Method. Methods. 2001;25:402‐408. 11846609 10.1006/meth.2001.1262

[35] Yan M , Liu X , Dang Q , Huang H , Yang F , Li Y . Intra‐articular injection of human synovial membrane‐derived mesenchymal stem cells in murine collagen‐induced arthritis: assessment of immunomodulatory capacity in vivo. Stem Cells Int. 2017;2017:9198328. 28751919 10.1155/2017/9198328 PMC5497673

[36] Rappold PM , Cui M , Grima JC , et al. Drp1 inhibition attenuates neurotoxicity and dopamine release deficits in vivo. Nat Commun. 2014;5:5244. 25370169 10.1038/ncomms6244 PMC4223875

[37] Song W , Chen J , Petrilli A , et al. Mutant huntingtin binds the mitochondrial fission GTPase dynamin‐related protein‐1 and increases its enzymatic activity. Nat Med. 2011;17:377‐382. 21336284 10.1038/nm.2313 PMC3051025

[38] Disatnik MH , Ferreira JC , Campos JC , et al. Acute inhibition of excessive mitochondrial fission after myocardial infarction prevents long‐term cardiac dysfunction. J Am Heart Assoc. 2013;2:e000461. 24103571 10.1161/JAHA.113.000461 PMC3835263

[39] Devi L , Prabhu BM , Galati DF , Avadhani NG , Anandatheerthavarada HK . Accumulation of amyloid precursor protein in the mitochondrial import channels of human Alzheimer's disease brain is associated with mitochondrial dysfunction. J Neurosci. 2006;26:9057‐9068. 16943564 10.1523/JNEUROSCI.1469-06.2006 PMC6675337

[40] Zepeda R , Kuzmicic J , Parra V , et al. Drp1 loss‐of‐function reduces cardiomyocyte oxygen dependence protecting the heart from ischemia‐reperfusion injury. J Cardiovasc Pharmacol. 2014;63:477‐487. 24477044 10.1097/FJC.0000000000000071

[41] Patrushev MV , Mazunin IO , Vinogradova EN , Kamenski PA . Mitochondrial fission and fusion. Biochem. 2015;80:1457‐1464. 26615436 10.1134/S0006297915110061

[42] Gan X , Huang S , Yu Q , Yu H , Yan SS . Blockade of Drp1 rescues oxidative stress‐induced osteoblast dysfunction. Biochem Biophys Res Commun. 2015;468:719‐725. 26577411 10.1016/j.bbrc.2015.11.022 PMC4834976

[43] Cassidy‐Stone A , Chipuk JE , Ingerman E , et al. Chemical inhibition of the mitochondrial division dynamin reveals its role in Bax/Bak‐dependent mitochondrial outer membrane permeabilization. Dev Cell. 2008;14:193‐204. 18267088 10.1016/j.devcel.2007.11.019 PMC2267902

[44] Ferrari LF , Chum A , Bogen O , Reichling DB , Levine JD . Role of Drp1, a key mitochondrial fission protein, in neuropathic pain. J Neurosci. 2011;31:11404‐11410. 21813700 10.1523/JNEUROSCI.2223-11.2011 PMC3157245

[45] Brooks C , Wei Q , Cho SG , Dong Z . Regulation of mitochondrial dynamics in acute kidney injury in cell culture and rodent models. J Clin Invest. 2009;119:1275‐1285. 19349686 10.1172/JCI37829 PMC2673870

[46] Ong SB , Subrayan S , Lim SY , Yellon DM , Davidson SM , Hausenloy DJ . Inhibiting mitochondrial fission protects the heart against ischemia/reperfusion injury. Circulation. 2010;121:2012‐2022. 20421521 10.1161/CIRCULATIONAHA.109.906610

[47] Bordt EA , Clerc P , Roelofs BA , et al. The putative Drp1 inhibitor mdivi‐1 is a reversible mitochondrial complex I inhibitor that modulates reactive oxygen species. Dev Cell. 2017;40(6):583‐594.e6. 28350990 10.1016/j.devcel.2017.02.020 PMC5398851

[48] Vaamonde‐Garcia C , Riveiro‐Naveira RR , Valcarcel‐Ares MN , Hermida‐Carballo L , Blanco FJ , Lopez‐Armada MJ . Mitochondrial dysfunction increases inflammatory responsiveness to cytokines in normal human chondrocytes. Arthritis Rheum. 2012;64:2927‐2936. 22549761 10.1002/art.34508

[49] Camps M , Ruckle T , Ji H , et al. Blockade of PI3Kgamma suppresses joint inflammation and damage in mouse models of rheumatoid arthritis. Nat Med. 2005;11:936‐943. 16127437 10.1038/nm1284

[50] An J , Wang X , Guo P , Zhong Y , Zhang X , Yu Z . Hexabromocyclododecane and polychlorinated biphenyls increase resistance of hepatocellular carcinoma cells to cisplatin through the phosphatidylinositol 3‐kinase/protein kinase B pathway. Toxicol Lett. 2014;229:265‐272. 24960055 10.1016/j.toxlet.2014.06.025

[51] Factor V , Oliver AL , Panta GR , Thorgeirsson SS , Sonenshein GE , Arsura M . Roles of Akt/PKB and IKK complex in constitutive induction of NF‐kappaB in hepatocellular carcinomas of transforming growth factor alpha/c‐myc transgenic mice. Hepatology. 2001;34:32‐41. 11431731 10.1053/jhep.2001.25270

